# There Is an ‘Unconscious,’ but It May Well Be Conscious

**DOI:** 10.5964/ejop.v13i3.1388

**Published:** 2017-08-31

**Authors:** Bernardo Kastrup

**Affiliations:** a Independent Scholar, Veldhoven, The Netherlands; Webster University Geneva, Geneva, Switzerland; University of Neuchâtel, Neuchâtel, Switzerland

**Keywords:** consciousness, co-consciousness, meta-consciousness, neural correlates of consciousness, unconscious, self-reflection, re-representation, dissociation, dissociative identity disorder, philosophy of psychology

## Abstract

Depth psychology finds empirical validation today in a variety of observations that suggest the presence of causally effective mental processes outside conscious experience. I submit that this is due to misinterpretation of the observations: the subset of consciousness called “meta-consciousness” in the literature is often mistaken for consciousness proper, thereby artificially creating space for an “unconscious.” The implied hypothesis is that all mental processes may in fact be conscious, the appearance of unconsciousness arising from our dependence on self-reflective introspection for gauging awareness. After re-interpreting the empirical data according to a philosophically rigorous definition of consciousness, I show that two well-known phenomena corroborate this hypothesis: (a) experiences that, despite being conscious, aren’t re-represented during introspection; and (b) dissociated experiences inaccessible to the executive ego. If consciousness is inherent to all mentation, it may be fundamental in nature, as opposed to a product of particular types of brain function.

The foundational theoretical inference of the clinical approach called “depth psychology”—whose origins can be traced back to the works of Frederic Myers, Pierre Janet, William James, Sigmund Freud and Carl Jung—is that the human psyche comprises two main parts: a conscious and an unconscious segment (Kelly et al., 2009, pp. 301-334). The conscious segment comprises mental activity to which one has introspective access. The so-called “ego” is the felt sense of personal self that arises in association with a subset of this introspectively-accessible activity—e.g. some bodily sensations, images, thoughts, beliefs, etc.—and it is in this sense that I use the word ‘ego’ throughout this paper. In contrast, the unconscious segment comprises mental activity to which one has no introspective access. Inaccessible as it may be, depth psychologists contend that mental activity in the “unconscious”—a term often used as a noun—still can and does influence one’s conscious thoughts, feelings and behaviors. A more modern articulation of the notion of a *mental* unconscious—as opposed to what has historically been called “unconscious cerebration” (Kelly et al., 2009, pp. 340-352)—can be found in the writings of Kihlstrom (1997), for example.

Recent empirical results seem to corroborate the hypothesis of a mental unconscious by revealing the presence of mental activity individuals cannot access through introspection, but which nonetheless causally conditions the individuals’ conscious thoughts, feelings and behaviors (e.g. Westen, 1999; Augusto, 2010; Eagleman, 2011). Hassin (2013) goes as far as insisting, “unconscious processes can carry out every fundamental high-level function that conscious processes can perform” (p. 196). He reviews empirical evidence indicating that the unconscious is capable of cognitive control, the pursuit of goals, information broadcasting and even reasoning (ibid., pp. 197-200). This echoes Dijksterhuis and Nordgren (2006), whose experiments indicate that the unconscious can encompass “all psychological phenomena associated with thought, such as choice, decision making, attitude formation and attitude change, impression formation, diagnosticity, problem solving, and creativity” (p. 96). Even practitioners of cognitive therapy, who have traditionally ignored the unconscious, have more recently found clinical value in interpreting possible indirect manifestations of inaccessible mental activity in the form of dreams (Rosner, Lyddon, & Freeman, 2004). This new scientific approach to the hypothesis of an unconscious has been called “the new unconscious” (Hassin, Ulleman, & Bargh, 2005).

Clearly, there is significant evidence for the presence of causally-effective mental activity that we ordinarily cannot access through introspection. The question, however, is whether mental activity inaccessible through introspection is necessarily *unconscious*. It is true that, from the perspective of clinical psychology, these two modalities are operationally indistinguishable, since the clinicians’ sole gauge of their patients’ range of consciousness is the patients’ own introspective reports. However, from a theoretical standpoint, it is conceivable that mental activity the ego cannot access through introspection could still be conscious, in the sense of being phenomenally experienced somewhere in the psyche. If so, this has significant implications for our understanding of the nature of consciousness—and of its relationship to brain function—in the fields of neuropsychology, neuroscience and philosophy of mind.

Indeed, although the conflation between lack of introspective access and lack of consciousness is operationally justifiable in a clinical setting, the widespread use of the qualifier ‘unconscious’ today suggests an intrinsic dichotomy in the nature of mental processes: some supposedly *aren’t* experienced whilst others, somehow, are. This implies that consciousness is not fundamental to mentation, but a property that emerges from particular arrangements or configurations of neurons. Primed and driven by this assumption, significant resources are spent in neuropsychology and neuroscience today in an effort to figure out what these arrangements or configurations are. Hypotheses currently under investigation vary from vast topologies of information integration across neurons (Tononi, 2004) to microscopic quantum processes within neural microtubules (Hameroff, 2006).

The present paper, on the other hand, elaborates on the possibility that these efforts are misguided, for introspectively-inaccessible mental processes may still be conscious: they may be phenomenally experienced in a manner—or in an area of the psyche—that escapes egoic introspection. This way, the notion of an unconscious, despite the broad use and influence of the term in today’s psychology, may at root be a linguistic inaccuracy originating from mere operational convenience. If so, then consciousness may not be the product of specific arrangements or configurations of neural activity, but a fundamental property of *all* mentation. The implications of this possibility for neuropsychology, neuroscience and philosophy of mind are hard to overestimate.

## Defining and Gauging Consciousness

Before we can meaningfully discuss unconsciousness—the alleged lack of consciousness—we must, of course, have clarity regarding the meaning of the word ‘consciousness.’ What does it mean to say that a mental process is conscious? In this paper, I shall use a rigorous definition well-accepted in neuropsychology, neuroscience and philosophy of mind: mental activity is *conscious* if, and only if, there is something—*anything*—it is like to have such mental activity in and of itself (Chalmers, 2003; Nagel, 1974). (A less rigorous but more easily understandable formulation of this definition is this: mental activity is conscious if there is something it *feels* like to have such mental activity in and of itself. The verb ‘to feel,’ however, is too ambiguous to be used in a rigorous definition, so philosophers of mind have reached consensus around the formulation I originally proposed above.) This way, if mental activity is *un*conscious, then there is nothing it is like to have such activity in and of itself, even if it, in turn, causes or influences conscious activity. Notice that this definition of consciousness honors our intuitive understanding of the word: you only consider yourself conscious right now because there is something it is like to be you while you read this paper. Otherwise, you would necessarily be unconscious.

To remain consistent with our intuitive understanding of words, I shall also say that mental activity corresponds to *experience* if, and only if, it is conscious. You experience reading this paper because you are conscious of it right now. If you were not, what sense would there be in saying that you experience it?

According to these definitions, higher-order thought (as defined in Schooler, 2002, p. 340) is unnecessary for there to be consciousness. The presence of the mere qualities of raw experience—which philosophers of mind call *qualia*—is already sufficient for a mental process to be considered conscious. In this context, the categorization proposed by Schooler (2002) is helpful: he distinguishes between “non-conscious (unexperienced), conscious (experienced), and meta-conscious (re-represented)” mental processes (p. 339). Only the latter entails higher-order thought.

Now notice that *direct* insight into one’s conscious inner life is limited to those experiences one’s ego can access through introspection and then report to self or others. In the words of Klein (2015), “It is *only* in virtue of knowledge by acquaintance that we know our mental states. … Accordingly, the use of introspective reports as a reliable and informative source of information about mental states has seen a resurgence over the past few decades” (p. 361, original emphasis). For this reason, the study of the Neural Correlates of Consciousness (NCCs) still largely consists in correlating objective measurements of neural activity with introspective assessments (Koch, 2004): patterns of neural activity accompanied by reported experience are considered NCCs. Indeed, as Newell and Shanks recently wrote (2014), “Whereas issues about how to define and measure awareness were once highly prominent and controversial, it now seems to be generally accepted that awareness should be operationally defined as reportable knowledge” (p. 15).

The problem is that, as I shall shortly elaborate upon, for the subject’s ego to access and report an experience there must be: (a) an associative link between the ego and the experience; and (b) a meta-conscious re-representation of the experience. Therefore, while subjects can report non-dissociated meta-conscious processes, *they fundamentally cannot distinguish between truly unconscious processes and conscious processes that simply aren’t meta-conscious*, for *both* types are equally unreportable to self and others. This is an alarming conclusion, for much of the work indicating the presence of an unconscious is based on (the lack of) introspective reports of experience. The next two sections expand on all this.

In what follows, I shall assume that introspective reports are as good as “reliable, relevant, immediate, and sensitive” (Newell & Shanks, 2014, p. 3). This is charitable towards the hypothesis of an unconscious, for—as Newell and Shanks (2014) argued —much of the evidence behind this hypothesis can be attributed to methodological artifacts: delayed introspective assessments leading to impaired recall, experimenters not providing sufficient opportunity for subjects to report the introspective insights they actually have, cross-task confusion, etc. My goal is to show that, *even if* the research underpinning the existence of an unconscious were free of methodological artifacts, there would *still* be compelling reasons to posit that mental processes unaccompanied by introspective reports of experience can be conscious nonetheless.

## Non-Self-Reflective Experiences

To gain introspective access to an experience it is not enough to merely have the experience; we must also consciously know *that* we have it. After all, what introspective insight could we gain about an experience of which we are not explicitly aware? Schooler (2002) elaborates:

Critical to both the centrality of the conscious/non-conscious distinction, and its equation with reportability, is the assumption that people are explicitly aware of their conscious experiences. However, this assumption is challenged when subjective experience is dissociated from the explicit awareness of that experience. Such dissociations demonstrate the importance of distinguishing between consciousness and ‘meta-consciousness.’ (p. 339.)

The conscious knowledge *of* the experience—which comes in addition to the experience itself—is what Schooler (2002) calls a “re-representation”:

Periodically *attention* is directed towards explicitly assessing the contents of experience. The resulting meta-consciousness involves an explicit *re-representation* of consciousness in which one interprets, describes, or otherwise characterizes the state of one’s mind. (pp. 339-340, emphasis added).

Although re-representation is necessary for introspection, it is largely absent, for instance, in dreams (Windt & Metzinger, 2007). This demonstrates compellingly that mental activity does *not* need to be re-represented in order to be experienced—after all, who can seriously doubt that dreams are experienced?—but only to be introspectively accessed. During ordinary dreams we simply experience, without consciously knowing *that* we experience.

More formally, suppose that one has an experience *X*. To gain introspective access to *X* one must have conscious knowledge *N* of *X*. But *N*—the “re-representation”—is a separate experience in its own right. One experiences the *knowing of X* as a quality closely related to, but distinct from, *X* itself. *N* is not encompassed, entailed or implied by *X*. Indeed, Schooler (2002) highlights the fact that re-representations can even *mis*represent the original experiences:

Once meta-consciousness is triggered, translation dissociations can occur if the re-representation process misrepresents the original experience. Such dissociations are particularly likely when one *verbally* reflects on non-verbal experiences or attempts to take stock of ambiguous or subtle perceptual experiences. (p. 340, emphasis added.)

To make these abstract considerations more concrete, consider your breathing right now: the sensation of air flowing through your nostrils, the movements of your diaphragm, the inflation and deflation of your lungs, etc. Were you *not* experiencing these sensations a moment ago, before I directed your attention to them? Or were you just unaware *that* you were experiencing them all along? By directing your attention to these sensations, did I make them *conscious* or did I simply cause you to experience the *extra* quality of knowing *that* the sensations were conscious? Clearly, even waking experiences can occur without re-representation.

Re-representations are the product of a self-reflective *configuration of* consciousness, whereby the latter turns in upon itself so to objectify its own contents (Kastrup, 2014, pp. 104-110). In humans, this usually occurs through the use of “semiotic mediation” (Valsiner, 1998), which is our ability to re-represent our experiences by *naming* them explicitly or implicitly. Gillespie (2007) gives an example: “In order to obtain dinner one must first name … one’s hunger … This naming, which is a moment of self-reflection, is the first step in beginning to construct, semiotically, a path of action that will lead to dinner” (p. 678).

Naturally, nothing prevents experiences from occurring outside the field of self-reflection—that is, occurring without being explicitly or implicitly named. Nixon (2010, p. 216), for instance, calls these “unconscious experiences,” which in my view is an oxymoron but illustrates the subtlety of the point. He lists several examples: blindsight (Stoerig & Cowey, 1997), prosopanosognosia (Sacks, 1985), sleepwalking, post-hypnotic suggestion, etc. Indeed, the emergence of so-called “no-report paradigms” in contemporary neuroscience attests to the abundant presence of waking experiences that are unreportable because they fall outside the field of self-reflection (Tsuchiya, Wilke, Frässle, & Lamme, 2015; Vandenbroucke, Fahrenfort, Sligte, & Lamme, 2014).

**Figure 1:**
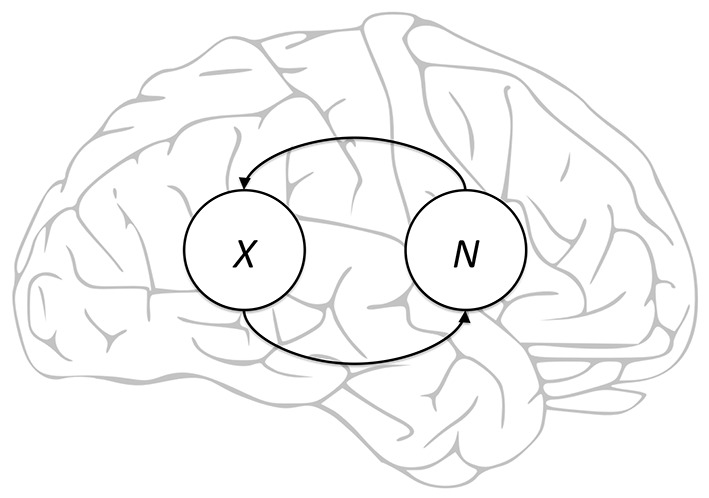
Illustrative caricature of oscillatory evocations between an experience (*X*) and the meta-conscious knowledge of the experience (*N*).

Moreover, *the neural activity patterns of the NCCs themselves* suggest circumstantially—yet compellingly—that many NCCs correspond merely to a self-reflective configuration of consciousness. To see this, notice first that the conscious knowledge *N* of an experience *X* is triggered by the occurrence of *X*. For instance, it is the occurrence of a sense perception that triggers the realization that one is perceiving something. *N*, in turn, evokes *X* by directing attention back to it: the realization that one is perceiving something naturally shifts one’s mental focus back to the original perception. So we end up with a back-and-forth cycle of evocations whereby *X* triggers *N*, which in turn evokes *X*, which again triggers *N*, and so forth. See Figure 1 for an illustration.

As it turns out, recent characterizations of the NCCs show precisely this pattern of reverberating back-and-forth communications between different brain regions (Boly et al., 2011; Dehaene & Changeux, 2011; van Gaal, Lamme, Fahrenfort, & Ridderinkhof, 2011). When damage to the primary visual cortex presumably interrupts this reverberation, patients display blindsight (Paller & Suzuki, 2014, p. 387)—that is, the ability to correctly discriminate moving objects despite the *reported* inability to see them. This is precisely what one would expect if the reverberation in question were the oscillations between *X* and *N*: the objects *are* consciously perceived—therefore explaining how the patients can discriminate them—but the patients do not know *that* they consciously perceive the objects.

I thus submit that many NCCs are, in fact, the correlates only of a potentially very small subset of consciousness—namely, meta-consciousness or self-reflection—instead of consciousness proper. The introspectively inaccessible character of experience that isn’t re-represented constitutes the first mechanism through which seemingly unconscious mental activity may, in fact, be conscious. There is yet another mechanism, which will be explored in the next section.

## Dissociated Experiences

Dissociative states are well recognized in psychiatry today, featuring prominently in the DSM-V (American Psychiatric Association, 2013). Their hallmark is “a disruption of and/or discontinuity in the normal integration of consciousness, memory, identity, emotion, perception, body representation, motor control, and behavior” (Black & Grant, 2014, p. 191). In other words, dissociation entails fragmentation of the contents of consciousness.

There are different forms of dissociation. Klein (2015), for instance, discusses a form in which the subject’s ego loses the sense of ownership of some of the subject’s own mental states. This occurs when consciousness can no longer “relate to its object in a particular, self-referential way” (p. 362). He lists several examples, such as the case of a man who, after an accident, could accurately report the content of his memories but “was unable to experience that content as his own” (p. 368). Notice, however, that the man’s ego could *still* access the content; just not identify with it.

In what follows, I shall focus on a strong form of dissociation in which the ego *cannot even access* certain contents of consciousness. In its pathological variations, this is known as Dissociative Identity Disorder (DID). A person suffering from DID exhibits multiple, disjoint centers of consciousness called alters. Each alter experiences the world as a distinct personality (Braude, 1995).

Although there has been debate about the authenticity of DID as a psychiatric condition—after all, it is conceivable that patients could fake it—research has confirmed DID’s legitimacy (Kelly et al., 2009, 167-174 & 348-352). Two recent studies are particularly interesting to highlight. In 2015, doctors reported on the case of a German woman who exhibited a variety of alters (Strasburger & Waldvogel, 2015). Peculiarly, some of her alters claimed to be blind while others could see normally. Through EEGs, the doctors were able to ascertain that the brain activity normally associated with sight wasn’t present while a blind alter was in control of the woman’s body, even though her eyes were open. When a sighted alter assumed executive control, the usual brain activity returned. This is a sobering result that shows the literally *blinding* power of dissociation. In another study (Schlumpf et al., 2014), investigators performed functional magnetic resonance imaging (fMRI) brain scans on both DID patients and actors simulating DID. The scans of the actual patients displayed clear and significant differences when compared to those of the actors. Undoubtedly, thus, DID is real.

Normally, only one of the alters has executive control of the body at any given moment. The important question for the purposes of the present paper is then this: Can the *other* alters, who are *not* in control of the body, remain conscious or do they simply fade into unconsciousness? If they can remain conscious, the implication is that a person can have multiple *concurrent* but dissociated centers of *consciousness*, as originally hypothesized by Frederic Myers and Pierre Janet (Kelly et al., 2009, pp. 305-317). Presumably, then, each center has its own private, parallel stream of experiences.

Occasionally, however, the dissociation isn’t bilateral: a first alter is able to gain partial access to the experiences of a second, without the second alter being able to access the experiences of the first. This rare kind of unilateral dissociation provides tantalizing indications that alters can remain conscious even when not in control of the body. In Morton Prince’s well-known study of the ‘Miss Beauchamp case’ of DID, one of the alters—called Sally—“was a co-conscious personality in a deeper sense. When she was not interacting with the world, she did not become dormant, but persisted and was active” (Kelly et al., 2009, p. 318). Sally maintained that she knew

everything Miss Beauchamp … does at the time she does it,—knows what she thinks, hears what she says, reads what she writes, and sees what she does; that she knows all this as a separate co-self, and that her knowledge does not come to her afterwards … in the form of a memory. (Prince, as quoted in Kelly et al., 2009, p. 318.)

Stephen Braude’s more recent work (1995) reinforces the view that alters can be co-conscious “discrete centers of self-awareness” (p. 67). He points—as evidence for this hypothesis—at the struggle of different alters for executive control of the body and the fact that alters “might intervene in the lives of others [i.e. other alters], intentionally interfering with their interests and activities, or at least playing mischief on them” (ibid., p. 68). It thus appears that alters can not only be concurrently conscious, but that they can also vie for dominance with each other.

Strong dissociation is not restricted to DID—its extreme form—or to pathology, for that matter. Indeed, the foundational hypothesis of depth psychology entails a form of natural dissociation between the conscious ego and the so-called “unconscious.” As such, it is plausible—in fact, there is overwhelming clinical evidence for it in the annals of depth psychology—that we all have at least one dissociated mental subsystem that we cannot access through introspection. Ernest Hilgard (1977) conceived of these dissociated subsystems as conscious, much as Myers, Janet and Braude did.

Thus, the possibility that presents itself to us is that we may all have one or more *conscious* ‘others’ within ourselves, dissociated from our ego. If this is so, then (a) our ego ordinarily has no introspective access to the experiences of these ‘others;’ and, consequently, (b) the study of the NCCs is largely blind to the potentially idiosyncratic patterns of neural activity corresponding to such dissociated experiences. This is the second mechanism through which apparently unconscious mental activity may, after all, be conscious.

## A Model of Dissociation

Wegner (2002) proposes an analogy for explaining alters: different operating systems running on the same hardware. This way, the transfer of executive control from one alter to another would be analogous to shutting down Windows and rebooting the computer with Linux. This, of course, only accounts for strictly alternating personalities and thus fails to explain much of the clinical data cited above. Nonetheless, it still suggests a starting point for a plausible model of dissociation.

**Figure 2:**
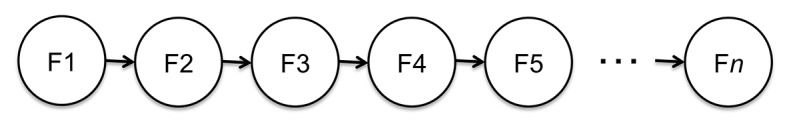
Conscious life as a chain of experiential frames connected through cognitive associations.

If we define an *experiential frame* as the set of all qualities we experience at a given moment—encompassing our conscious perceptions, thoughts, emotions, bodily sensations, imagination, etc.—conscious life can be modeled as a chain of experiential frames. This is graphically illustrated in Figure 2, wherein experiential frames F1 to F*n* are shown. Each frame is evoked by the previous frame through cognitive associations, in the sense that e.g. our particular thoughts in the present moment largely determine which emotions we experience in the next moment; or that our emotions in the present moment largely determine our actions—and therefore perceptions—in the next moment; and so on. These cognitive associations are represented by the arrows linking frames together in Figure 2.

**Figure 3:**
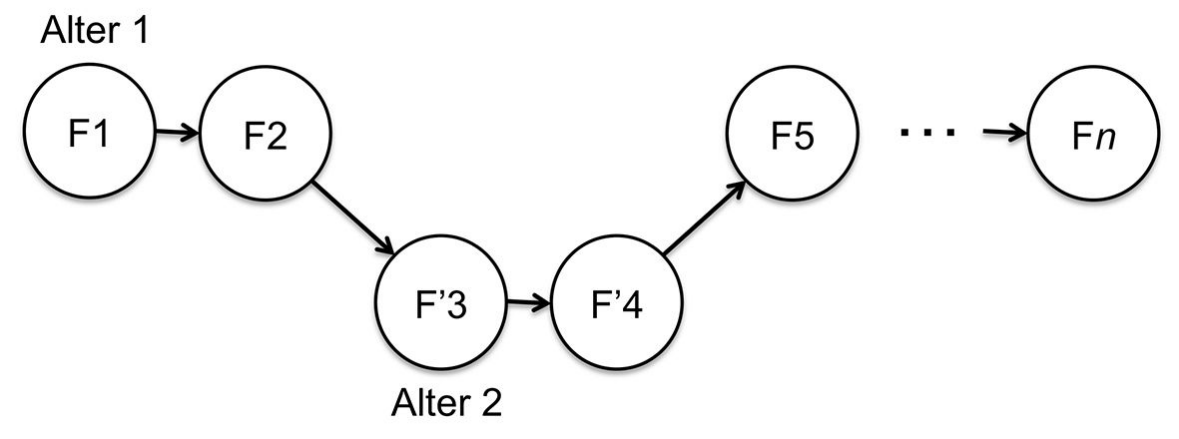
The sequential model of dissociation in the context of DID.

Wegner’s suggestion can then be visualized as in Figure 3. The chain of experiential frames—denoted F—corresponding to a first alter is interrupted by experiential frames—denoted F’—corresponding to a second alter. The key point is that, once executive control is assumed by the experiential frames F’ of the second alter, the corresponding experiential frames F of the first alter *cease to exist*. There is no parallelism of experience: either the mental contents of the first alter are experienced or those of the second alter; never those of both concurrently. As such, this is a *sequential model of dissociation* and, as we’ve seen, it isn’t sufficient to explain the clinical data cited.

**Figure 4:**
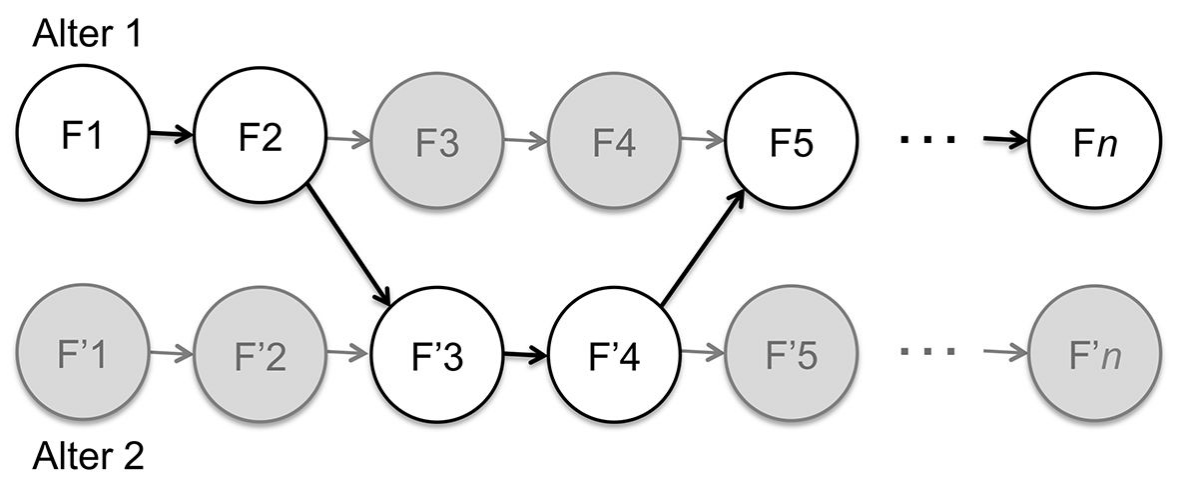
The parallel model of dissociation in the context of DID.

Alternatively, we can hypothesize that the chains of experiential frames of *both* alters are *always* present, concurrently and in parallel. Executive control of the body simply switches between the two parallel chains, as shown in Figure 4. Experiential frames drawn in grey represent those without executive control, *but still conscious*. This is thus a *parallel model of dissociation*, which illustrates the hypothesis of “co-consciousness” (a term originally coined by Morton Prince, as discussed by Kelly et al., 2009, p. 317).

**Figure 5:**
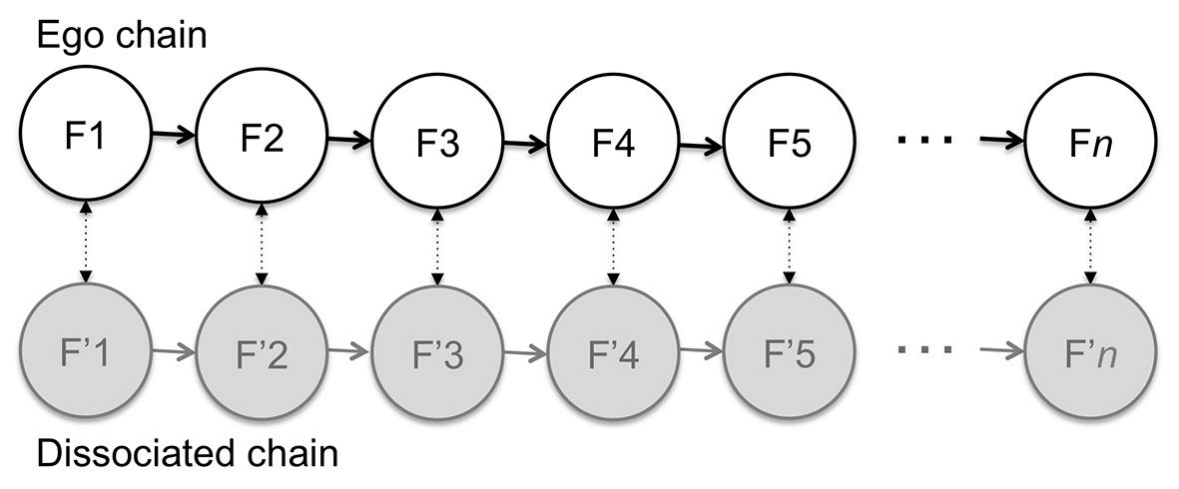
The parallel model of dissociation in a depth-psychological context.

We have seen that DID is a pathological form of dissociation, but that we may all naturally have strongly dissociated mental subsystems that never—or very seldom—vie for executive control of the body. These would constitute the so-called “unconscious” of depth psychology. Figure 5 illustrates how such strongly dissociated mental subsystems can be modeled under the proposed framework. For simplicity, only the ego and one dissociated subsystem are shown. The ‘other’ in this case—represented by the dissociated chain of experiential frames F’—is content to live its inner life in the background of egoic activity. It only manifests its presence through indirect, subtle influences on egoic experiences, as represented by the dashed arrows vertically linking the two chains. These subtle influences can take many forms, such as: dissociated emotions influencing our egoic thoughts and behaviors (Lynch & Kilmartin, 2013, p. 100); dissociated beliefs and expectations influencing our egoic perceptions (Eagleman, 2011, pp. 20-54); dissociated drives manifesting themselves symbolically in the form of dreams (Fonagy, Kächele, Leuzinger-Bohleber, & Taylor, 2012; Jung, 2002; von Franz & Boa, 1994); etc.

Admittedly, limitations in our ability to gauge consciousness currently prevent us from asserting with certainty, on an empirical basis, that the parallel model of dissociation is correct. However, by the same token, we can also not assert that it isn’t. The brain seems to have sufficient resources for this kind of parallelism and, if anything, the clinical data is suggestive of its validity (again, Kelly et al., 2009, pp. 305-322 and Braude, 1995). The parallel model should, therefore, be considered not only plausible but perhaps even probable, in which case it further substantiates the notion that the “unconscious” may be—well—conscious.

## Discussion

I have elaborated on the hypothesis that there may be no such a thing as an unconscious mental process. All mental processes may be conscious, in the sense that there may be something it is like to have such mental processes in and of themselves. Our impression that some mental processes are unconscious may arise from (a) their consisting in non-self-reflective experiences not amenable to introspection or (b) their being strongly dissociated from the executive ego and, therefore, inaccessible to it.

Underlying this entire paper is the differentiation between consciousness proper and particular *configurations of* consciousness, such as self-reflection and dissociative states. It is rather disturbing how often these notions are conflated not only in general psychology, but also in neuroscience and philosophy of mind. For instance, a *Scientific American Mind* article penned by neuroscientist Christof Koch (2009) uses the word ‘consciousness’ multiple times when it in fact refers to meta-consciousness. Whilst its title asks “When Does Consciousness Arise in Human Babies?” much of the discussion is centered on self-reflection.

Dijksterhuis and Nordgren (2006) also “define conscious thought as object-relevant or task-relevant cognitive or affective thought processes that occur while the object or task is *the focus of one’s conscious attention*” (p. 96, emphasis added). They insist, “it is very important to realize that *attention is the key* to distinguish [*sic*] between unconscious thought and conscious thought. *Conscious thought is thought with attention*” (ibid., emphasis added). In appealing to *attention*, as opposed to experience or *qualia*, they are implicitly associating consciousness with self-reflection or re-representation, as discussed in Section 3.

Even more strikingly, Cleeremans (2011) *explicitly defines* consciousness as self-reflection. He overtly conflates experience with meta-consciousness and reportability:

*Awareness*, on the other hand, always seems to minimally entail the ability of knowing *that* one knows. This ability, after all, forms the basis for the verbal reports we take to be the most direct indication of awareness. And when we observe the absence of such ability to report on the knowledge involved in our decisions, we rightfully conclude that the decision was based on unconscious knowledge. Thus, it is when an agent exhibits *knowledge* of the fact that he is sensitive to some state of affairs that we take this agent to be a conscious agent. This *second-order* knowledge, I argue, critically depends on *learned* systems of meta representations, and forms the basis for conscious experience. (p. 3.)

This isn’t a recent problem. When one reads the original texts of the founders of depth psychology whilst holding the distinction between consciousness and meta-consciousness in mind, one quickly realizes that, when they spoke of unconsciousness, the founders often meant a lack of *meta*-consciousness—not of experience proper. This is abundantly evident, for instance, in an essay written by Carl Jung in the 1920s or early 1930s, called “The Stages of Life” (Jung, 2001, pp. 97-116).

It could be argued that the distinction between experience and meta-consciousness is merely a semantic point. However, consider this: by conflating consciousness proper with *self-reflective* consciousness, we also indirectly equate non-self-reflective consciousness with unconsciousness; we absurdly imply that dreams—which largely lack self-reflection (Windt & Metzinger, 2007)—aren’t experienced. Instead of the three categories proposed by Schooler (2002)—namely, “non-conscious (unexperienced), conscious (experienced), and meta-conscious (re-represented)” (p. 339)—we are left with only two: non-conscious and meta-conscious. Consequently, we are forced to collapse the conscious onto the non-conscious and, in the process, end up disregarding the extraordinary phenomenon of *qualities of experience*. Clearly, this isn’t merely semantic.

Most importantly, the philosophical implications of mistaking consciousness for *meta*-consciousness are significant. If some mental processes were truly unconscious while others are conscious, it would follow that consciousness is the product of some specific anatomical and/or functional arrangements of brain activity. In other words, consciousness would be derivative, as opposed to fundamental. Philosophically, this would corroborate the ontology of physicalism (Stoljar, 2016) while contradicting alternatives like panpsychism (Strawson et al., 2006), cosmopsychism (Shani, 2015) and idealism (Kastrup, 2017). It would leave us with no way to circumvent the arguably insoluble “hard problem of consciousness” (Chalmers, 2003).

On the other hand, if consciousness is inherent to all mental processes, then the specific anatomical and/or functional parameters of different processes correspond merely to different *contents and/or configurations of consciousness*—that is, to the particular qualities that are experienced—but do not determine the presence or absence of consciousness itself. This allows us to circumvent the “hard problem of consciousness” altogether, by inferring that consciousness is primary. While it’s not my intent in this paper to argue for or against any particular ontology of mind, it is significant that a lucid, critical interpretation of the available empirical data leaves more avenues of philosophical inquiry open.

If we are true to the spirit of the words ‘consciousness’ and ‘experience,’ diligent in our interpretation of empirical observations—both experimental and clinical—and rigorous in our use of concepts, we are led not only to the conclusion that *all* mental processes may be conscious, but that consciousness itself may be fundamental.
